# Antimicrobial and Safety Properties of Lactobacilli Isolated from two Cameroonian Traditional Fermented Foods

**DOI:** 10.3797/scipharm.1107-12

**Published:** 2011-12-18

**Authors:** Pierre Marie Kaktcham, Ngoufack François Zambou, Félicité Mbiapo Tchouanguep, Morsi El-Soda, Muhammad Iqbal Choudhary

**Affiliations:** 1 Laboratory of Biochemistry, Food Science and Nutrition (LABPMAN), Department of Biochemistry, Faculty of Science, University of Dschang, P.O. Box 67 Dschang, Cameroon; 2 HEJ Research Institute of Chemistry, International Center for Chemical and Biological Sciences (ICCBS), University of Karachi, Karachi-75270, Pakistan; 3 Laboratory of microbial Biochemistry, Faculty of Agronomy, University of Alexandria, Egypt

**Keywords:** Sha’a, Kossam, *Lactobacillus spp*., Rep-PCR, Safety properties, Antimicrobial activity

## Abstract

Twenty-one *Lactobacillus* isolates from “*Sha’a*” (a maize – based fermented beverage) and “*Kossam*” (traditionally fermented cow milk) were selected in accordance with their antagonistic activities and tested for their bacteriocinogenic potential as well as safety properties. These isolates were preliminarily identified as *Lactobacillus plantarum* (62%), *Lactobacillus rhamnosus* (24%), *Lactobacillus fermentum* (10%) and *Lactobacillus coprophilus* (4%) based on phenotypic characteristics and rep-PCR genomic fingerprinting. Twelve (57.1%) out of the 21 strains tested were found to be bacteriocin producers, as revealed by the sensitivity of their antimicrobial substances to proteolytic enzymes (Trypsin, Proteinase K) and inhibition of other *Lactobacillus spp*. These bacteriocinogenic strains showed no positive haemolytic and gelatinase activities and proved to be sensitive to penicillin G, ampicillin, tetracycline, erythromycin, amoxicillin, chloramphenicol, co-trimoxazole and doxycyclin, but resistant to ciprofloxacin and gentamicin. The bacteriocins showed a broad inhibitory activity against Gram-positive and Gram-negative pathogenic bacteria, several of which are classified as especially dangerous by the World Health Organization, as well as Multidrug-resistant strains. These include *Staphylococcus aureus, Salmonella enterica* subsp. *enterica* serovare Typhi*, Bacillus cereus, Streptococcus mutans, Escherichia coli, Pseudomonas aeruginosa, Klebsiella pneumoniae* and *Shigella flexneri*. These *Lactobacillus* strains are promising candidates for use as protective cultures in food fermentation.

## Introduction

Lactic acid bacteria (LAB) have been used in the processing of fermented foods for centuries [1]. They are part of the daily diet of virtually all people around the world. Most often, production of indigenous fermented foods depends on naturally occurring LAB. They have the ability to produce a variety of antimicrobial substances such as organic acids, hydrogen peroxide and bacteriocins. Bacteriocins produced by LAB are ribosomally synthesized extracellular small peptides that exhibit bactericidal or bacteriostatic activity against genetically closely related bacteria [2]. They are the most important, because, due to their proteinaceous nature, they are rapidly digested by proteases in human and animal gastrointestinal tracts, unlike current antibiotics [3]. Given that the prevalence of multidrug-resistant (MDR) and especially dangerous pathogenic bacteria is increasing at an alarming rate [4], bacteriocins or bacteriocin-like substances could be a novel approach for an effective drug. In addition, fermented foods are also associated with LAB which possess remarkable properties for their use as starter cultures or probiotics. They are usually used for their health benefits in animal or human, and it is recommended that antibiotic resistance patterns and opportunistic virulence properties should be tested to document their safety [5]. In fact, antibiotic-resistant LAB could horizontally transfer their genes or determinants to opportunistic pathogens within the gut microbiota [6].

In the western highlands region of Cameroon, two traditional fermented foods named “*Kossam*” (fermented cow milk) and “*Sha’a*” (a maize-based beverage) are widely produced and consumed. While they constitute a natural reservoir of LAB which have not yet been identified and studied for their antimicrobial activity, there is obvious evidence that LAB strains from different origins could possess antimicrobial activities at different levels. It became important to initiate comprehensive studies to screen antimicrobial and safety properties of the wild LAB microbiota from these natural reservoirs for their antimicrobial applications. This research is important in order to valorise indigenous strains and also to propose another alternative to satisfy the increasing request of the market with novel bacteriocinogenic characterized LAB strains.

In the present study, lactobacilli with antagonistic activity isolated from “*Kossam*” and “*Sha’a*” were characterized and identified. They were also evaluated for bacteriocin production as well as some safety properties such as antibiotic susceptibility, haemolysis and gelatinase activities.

## Results and Discussion

### Isolation of Lactobacillus strains and inhibitory activity

A total of 90 *Lactobacillus* isolates was obtained. Sixty (75%) out of the 90 isolates inhibited the growth of the other lactobacilli strains, as well as *Staphylococcus aureus* ATCC25923, *Salmonella enterica* subsp. *enterica* serovare Typhi ATCC6539, *Pseudomonas aeruginosa* ATCC27853, *Escherichia coli*, *Klebsiella pneumoniae*, *Proteus mirabilis* and *Shigella flexneri*. From these 60 isolates, 21 (35%) with largest spectra and zone of inhibition were selected and used for further assays.

### Preliminary identification of strains

The microscopic examination reveals that the tested isolates have cellular rod form, associated in pairs, heap or chain. In addition, all the isolates were found to be gram-positive and catalase-negative. Based on the fermentative profile, data from each isolate was compared with profiles obtained from recognized test organisms, and it was determined that the isolates can be classified as *Lb. plantarum*, *Lb. rhamnosus*, *Lb. coprophilus* and *Lb. fermentum* (Table 1).

**Fig. 1:**
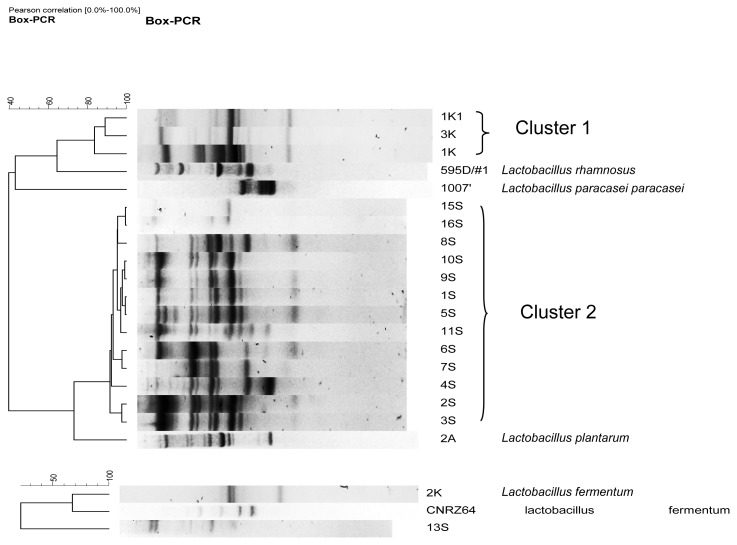
Dendrograms generated from Box-PCR fingerprinting of the isolated Group II and Group III *Lactobacillus spp*. The dendrogram was constructed using the unweighted pair group method using arithmetic averages with correlation levels expressed as percentage.

From the 21 isolates tested and identified using phenotypic characteristics, 18 were identified by rep-PCR. The dendrogram generated from BOXA1R-PCR banding patterns are given in Figure 1. The isolates belonging to group II *Lactobacillus spp.* were classified into 2 clusters. Cluster 1 consisted of *Lb. rhamnosus* (1K, 1K1, 3K) at r = 69.5%, whereas cluster 2 grouped *Lb. plantarum* strains (1S, 2S, 3S, 4S, 5S, 6S, 7S, 8S, 9S, 10S, 11S, 15S, 16S) at r = 73.06% (Figure 1).

The isolates belonging to the Group III *Lactobacillus spp* (13S, 2K) were similar to strains of *Lb. fermentum* at r = 68.8%. The strains of each cluster also showed characteristic banding pattern with slight differences, demonstrating their close relatedness. The majority of *Lactobacillus* strains with antagonistic activity was found in “*Sha’a*” where a predominance of *Lactobacillus plantarum* was observed, whereas in “*Kossam*”, *Lactobacillus rhamnosus* was predominant. This result is in accordance with recent investigations showing that LAB with antagonistic activities are largely distributed in cereal-based fermented foods [21, 22]. Rep-PCR confirms all phenotypic results, except for one strain (13S). After identification by API system this strain has higher percent similarity with *Lactobacillus coprophilus* than *Lactobacillus fermentum*, but it was identified as *Lactobacillus fermentum* by rep-PCR. Such situations were also found by authors such as Chagnaud *et al*. [23], Mohamed *et al*. [9] and Terzic-Vidojevic *et al.* [24]. This shows that phenotypic tests sometimes suffer from lack of reproducibility and discriminatory power.

### Screening for bacteriocin producing strains

**Fig. 2:**
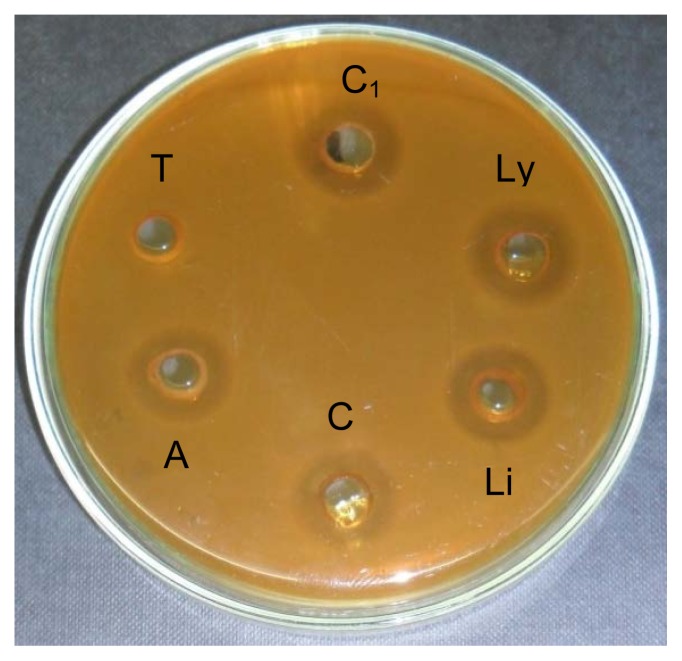
Antimicrobial activity assessed by halo formation of bacteriocin from strain *Lb. plantarum* 6S treated with enzymes. C_1_: CFS in Tris-HCl buffer, pH 8.0 (untreated). C: CFS in Phosphate buffer, pH 7.0 (untreated). T: CFS treated with Trypsin (1 mg/ml). A: CFS treated with α-Amylase (1 mg/ml). Li: CFS treated with Lipase (1 mg/ml). Ly: CFS treated with Lysozyme (1 mg/ml).

Using combination of Triple-agar layer method and AWDA, 12 (57.1%) out of the 21 strains tested were found to be bacteriocin producers. Complete inactivation was observed when the CFS were treated by proteolytic enzymes, thus confirming the proteinaceous nature of the inhibitory substances. Treatment with α-Amylase and Lipase did not affect the antimicrobial activity, suggesting that the bacteriocins are not attached to carbohydrate or lipid moieties (Table 2 and Figure 2).

Inhibition of sensitive strain of *Lactobacillus plantarum* confirmed that the inhibitory substances were bacteriocins. This study reports for the first time the characterization of bacteriocin-producing LAB isolated from “*Sha’a*” and traditionally fermented cow milk from Cameroon. Although many studies reported bacteriocin production by LAB worldwide, few strains of *Lactobacillus fermentum* were listed [25, 26].

### Safety attributes of bacteriocin-producing strains

All 12 strains assayed showed no positive haemolysis and gelatinase activity. With respect to haemolysis activity, these strains were found to be γ-haemolytic. Results of the antibiotic susceptibility of strains are listed in Tables 3 and 4.

Based on the disc diffusion test results, all the strains (100%) were susceptible to inhibitors of the cell wall synthesis (penicillin G, ampicillin and amoxicillin); they were also all susceptible to chloramphenicol and erythromycin. Sixty-six percent showed moderate susceptibility to tetracycline and doxycyclin, whereas 50% were resistant to gentamicin as far as inhibitors of protein or mRNA synthesis were concerned. The strains showed susceptibility (50%) and moderate susceptibility (50%) to co-trimoxazole, but were all resistant (100%) to ciprofloxacin, these antibiotics belonging to the group of nucleic acid synthesis’s inhibitors.

With regard to MIC results, the strains showed resistance (100%) only to gentamicin as revealed by the comparison with the EFSA’s breakpoints. According to the ISO’s epidemiological cut-off values, two out of the 10 *Lb. plantarum* strains were recorded with MICs for ciprofloxacin above the quality control (QC) range. For all the *Lb. rhamnosus* strains, only the MICs for gentamicin and ciprofloxacin were above the QC range. Safety is one of the recommended attributes in guidelines on evaluation for probiotics and other LAB to be used as food additives [5]. Haemolysis activity would break down the epithelial layer while gelatinase activity would derange the mucoid lining interfering with the normal functioning of these very important linings across which many physiological substances are exchanged and would cause pathways for infections. None of the strains showed haemolysis or gelatinase activity, and these results were in accordance with those of Kalui *et al.* [27] for *Lb. plantarum* strains*, Lb. rhamnosus* strains and *Enterococcus faecium* ET05. A key requirement for these food additives LAB strains is that they should not carry transferable antibiotic resistance genes. Transferable resistance genes may pose a risk, as they can be transferred to pathogenic bacteria [28]. From the 10 antibiotics tested, some strains were found to be resistant only to two of them, ciprofloxacin and gentamicin. Such cases were also reported by other authors such as Elkins and Mullins, [29]; Herreros *et al* [30]; Rojo-Bezares *et al.* [31] who found resistance of *Lactobacilli* to ciprofloxacin, gentamicin and other aminoglycosides. These resistances are natural and intrinsic resistances, probably due to cell wall structure and membrane impermeability, complemented in some cases by potential efflux mechanisms. Intrinsic resistance is not horizontally transferable and poses no risk in non-pathogenic bacteria [32]. Furthermore, following the EUCAST definition of an epidemiological value [20], our strains can be categorized as wild type organisms (free of acquired and mutational resistance mechanisms). Therefore, all the strains tested in our study are considered to be safe.

### Spectrum of Inhibitory activity

The inhibitory spectrum of the bacteriocins produced by selected strains is presented in Table 5.

The bacteriocins showed a relatively wide inhibition spectrum, inhibiting the growth of a number of Gram-positive and Gram-negative bacteria including species of the genera *Lactobacillus*, *Streptococcus*, *Salmonella*, *Shigella, Bacillus*, *Staphylococcus, Escherichia, Pseudomonas* and *Klebsiella*. However, no activity was detected against many other *Lactobacillus* strains and the *Enterococcus faecium* strain tested. Interestingly, some bacteriocins were active against multidrug-resistant (MDR) strains of *S. aureus* and *E. coli*. Multidrug-resistant bacteria have emerged as serious pathogens over the past decade and, despite major research efforts aimed at finding an effective drug, increasing resistance has compromised therapy [33].

## Experimental

### Samples, bacterial strains and growth conditions

Fifteen and 20 samples respectively of “*Kossam*” and “*Sha’a*” were collected directly and aseptically from local producers in three localities of Cameroon’s western highlands region. For lactic acid bacteria isolation, 1% (v/v) of each sample added to MRS broth (de Man Rogosa and Sharpe, Biolife, Milano, Italy) and incubated for 24 h at 30 °C was used to streak the surface of MRS agar plates. The plates were then incubated anaerobically (Genbox anaer; BioMérieux, France) at 30 °C for 48 h. Well-developed individual colonies on these plates were randomly picked and purified on MRS agar. These isolates were characterized based on the morphological characteristics, gram staining and catalase reaction [7]. The lactobacilli strains were kept in MRS broth plus glycerol (70:30) at −20 °C and were subcultured two times in MRS broth for activation prior to experimental use.

The reference strains for Rep – PCR analysis were obtained from Institut National de Recherche Agronomique (INRA), Centre de Recherche Zootechnique Jouy – en Josas, France (CNRZ) and American Type Culture Collection (ATCC).

Food borne spoilage and pathogenic bacteria (*Proteus mirabilis* and those listed in Table 5) used as indicator in testing antimicrobial activity were cultured at 37 °C and maintained on Mueller Hinton Agar (MHA, Conda, Madrid, Spain) slants.

### Inhibition of the growth of other lactobacilli and pathogenic bacteria

The LAB isolates were tested for inhibition of the growth of other lactobacilli and some pathogenic bacteria using the spot on the lawn test as described by Geis *et al.* [8]. An aliquot (2 μl) of an overnight LAB culture was spotted onto MRS agar plates and incubated anaerobically at 30 °C for 48 h. The plates were subsequently overlaid with soft MRS agar (0.75% agar) or soft MHA containing 1% indicator strains, respectively, and then incubated anaerobically at 30 °C or aerobically at 37 °C, on the basis of the tested organisms, for 24 h. Only isolates showing the largest inhibition diameter zones were selected for the next steps.

### Strains identification using physiological and biochemical methods

The 21 selected isolates were identified to species level using their carbohydrate pattern obtained with API 50 galleries (BioMérieux, Marcy l’Etoile France). Tests were performed according to the manufacturer’s instructions. Interpretations of the fermentation profiles were facilitated by systematically comparing all results obtained from the isolates studied with information from the computer-aided database APILAB plus V.3.2.2.

### Rep-PCR genomic fingerprinting

Total DNA was extracted from 1.6 ml of fresh cultures in the exponential growth phase using the Wizard DNA purification Kit as described by the manufacturer (Promega). Amplification of DNA and separation of PCR products were performed according to the method described by Mohammed *et al.* [9]. The Rep profiles were processed using the Gel Compar version 5.00 software (Applied Maths, Kortrijk, Belgium).

### Screening of Lactobacillus strains for bacteriocin production

The *Lactobacillus* strains were screened for bacteriocin production using the Triple-agar layer method described by Todorov and Dicks [10], with the difference that buffered MRS medium (0.2 M potassium phosphate buffer, pH 7.0) was used and no antibiotic was added.

Cell-free supernatants (CFS) of the selected producer strains were screened for bacteriocin activity by the agar well diffusion assay (AWDA) as described by Schillinger and Lücke [11]. A 15-h-old culture (2% v/v) of each lactobacilli strain was inoculated in buffered MRS broth and incubated anaerobically at 30 °C for 10 h. The cultures were centrifuged (7,000 rpm, 30 min, 4 °C) and the supernatants collected, treated at 80 °C for 10 min [12] and then tested for their activity by the AWDA. In another set of experiments, the CFS were incubated for 2 h at 37 °C in the presence of 1.0 mg/ml (final concentration) of Trypsin (in 0.05 M Tris-HCl buffer, pH 8.0, Fluka Biochemika), Proteinase K (in 0.05 M phosphate buffer, pH 7.0, Merck) α-Amylase (in 0.05 M phosphate buffer, pH 7.0, Sigma-Aldrich), Lipase (in 0.05 M phosphate buffer, pH 7.0, Sigma-Aldrich) and Lysozyme (in 0.05 M phosphate buffer, pH 7.0, Fluka Biochemika) and then tested for antimicrobial activity by AWDA.

### Safety attributes of bacteriocin-producing strains

The safety attributes studied were haemolytic activity, gelatinase activity and antibiotic susceptibility.

Haemolytic activity was investigated as described by Gerhardt *et al.* [13]. A 16-hour-old broth culture was streaked onto sterile blood agar plates. Plates were incubated anaerobically at 30 °C for 48 h. The haemolytic reactions were recorded by observation of a clear zone around the colonies (β-haemolysis), a partial hydrolysis and greening zone (α-haemolysis) or no reaction (γ-haemolysis) [14].

Gelatinase activity was investigated as described by Harrigan and Mc Cance [15]. A 16-hour-old culture was streaked onto nutrient gelatin agar (Oxoid). The plates were incubated anaerobically for 48 h at 30 °C after which they were flooded with a saturated ammonium sulfate solution and observed for clear zones surrounding colonies.

Antibiotic susceptibility was tested by disk diffusion and by broth micro-dilution methods [16–17], using LSM broth and agar as test media (LSM consists of a mixture of Iso-Sensitest medium, Oxoid Ltd). Incubation was done at 30 °C for 48 h. The MIC (μg/ml) was defined as the lowest antibiotic concentration that resulted in no visible growth. For disc diffusion antibiotic susceptibility, inhibition zone diameters (IZD) were measured and strains were classified as sensitive (IZD ≥ 21mm), intermediate (16mm ≤ IZD ≤ 20mm), and resistant (IZD ≤ 15mm) according to interpretative standards defined by CLSI [18] and Vlková *et al.* [19]. The MICs (μg/ml) were determined and the results of susceptibility status were interpreted according to the recent FEEDAP document of the European Food Safety Authority (EFSA) on the update of the criteria used in the assessment of antibiotics bacterial resistance of human or veterinary importance [5] as well as epidemiological cutoff values defined by the ACE – ART Project results, ISO 10932 [20]. Strains showing MICs less than EFSA’s breakpoints were considered sensitive; otherwise, they were resistant. The following antibiotics obtained from Oxoid were tested: penicillin G, ampicillin, ciprofloxacin, tetracycline, erythromycin, amoxicillin, gentamicin, chloramphenicol, co-trimoxazole, and doxycyclin.

### Spectrum of inhibitory activity of bacteriocin-producing strains

The antibacterial activities of the samples were tested against Gram-positive and Gram-negative bacteria (listed in table 5). The indicator strains (0.5 Mc Farland suspensions) were inoculated in the appropriate soft agar media and the antibacterial activities were determined by AWDA. Experiments were conducted in triplicate.

## Figures and Tables

**Tab. 1:** Comparison of the identification of Lactobacilli isolates using API system and rep-PCR profiles.

Isolates	Identification by API system (% similarity)	Identification by rep-PCR (% identity)
**1S**	*Lactobacillus plantarum (99.9%)*	*Lactobacillus plantarum (72.5%)*
**2S**	*Lactobacillus plantarum (99.9%)*	*Lactobacillus plantarum (80.0%)*
**3S**	*Lactobacillus plantarum (99.9%)*	*Lactobacillus plantarum (75.8%)*
**4S**	*Lactobacillus plantarum (75.9%)*	*Lactobacillus plantarum (74.6%)*
**5S**	*Lactobacillus plantarum (99.9%)*	*Lactobacillus plantarum (74.9%)*
**6S**	*Lactobacillus plantarum (99.9%)*	*Lactobacillus plantarum (74.5%)*
**7S**	*Lactobacillus plantarum (99.9%)*	*Lactobacillus plantarum (72.6%)*
**8S**	*Lactobacillus plantarum (99.9%)*	*Lactobacillus plantarum (73.0%)*
**9S**	*Lactobacillus plantarum (72.0%)*	*Lactobacillus plantarum (72.9%)*
**10S**	*Lactobacillus plantarum (79.0%)*	*Lactobacillus plantarum (73.9%)*
**11S**	*Lactobacillus plantarum (77.0%)*	*Lactobacillus plantarum (73.6%)*
**15S**	*Lactobacillus plantarum (85.9%)*	*Lactobacillus plantarum (65.8%)*
**16S**	*Lactobacillus plantarum (89.9%)*	*Lactobacillus plantarum (65.7%)*
**18S**	*Lactobacillus rhamnosus (97.5%)*	*Unidentified*
**19S**	*Lactobacillus rhamnosus (86.0%)*	*Unidentified*
**1K**	*Lactobacillus rhamnosus (86.0%)*	*Lactobacillus rhamnosus (73.1%)*
**3K**	*Lactobacillus rhamnosus (88.5%)*	*Lactobacillus rhamnosus (65.0%)*
**1K1**	*Lactobacillus rhamnosus (96.5%)*	*Lactobacillus rhamnosus (70.4%)*
**2K**	*Lactobacillus fermentum (74.1%)*	*Lactobacillus fermentum (69.2%)*
13S*	*Lactobacillus coprophilus (77.0%)*	*Lactobacillus fermentum (68.4%)*
**1SB1**	*Lactobacillus coprophilus (97.0%)*	*unidentified*

* …API result does not match with rep-PCR identification in this case.

**Tab. 2:** Effect of proteolytic and non-proteolytic enzymes on the activity of cell free supernatants.

	Mean Inhibition Zone diameter (mm)^a^					

Strains	Control	Trypsin	Proteinase K	α-Amylase	Lipase	Lysozyme
*Lb. plantarum* 2S	12.5	0 (−)	0 (−)	12 (+)	12.5 (+)	12.5 (+)
*Lb. plantarum* 5S	13.5	0 (−)	0 (−)	13.5 (+)	13.5 (+)	13.5 (+)
*Lb. plantarum* 6S	16	0 (−)	0 (−)	15.5 (+)	15.5 (+)	16 (+)
*Lb. plantarum* 7S	13	0 (−)	0 (−)	12.5 (+)	12.5 (+)	12.5 (+)
*Lb. plantarum* 8S	12.5	0 (−)	0 (−)	12.5 (+)	12.5 (+)	12.5 (+)
*Lb. plantarum* 9S	13	0 (−)	0 (−)	13 (+)	13 (+)	13 (+)
*Lb. pantarum* 10S	8	0 (−)	0 (−)	8 (+)	8 (+)	8 (+)
*Lb. plantarum* 11S	13	0 (−)	9 (+)	13 (+)	13 (+)	13 (+)
*Lb. plantarum* 16S	12	0 (−)	0 (−)	12 (+)	12 (+)	12 (+)
*Lb. rhamnosus* 18S	12	0 (−)	0 (−)	12 (+)	12 (+)	12 (+)
*Lb. rhamnosus* 1K	14	0 (−)	0 (−)	14 (+)	14 (+)	14 (+)
*Lb. fermentum* 2K	13	0 (−)	0 (−)	13 (+)	13 (+)	13 (+)

a …Inhibition zone diameters are means of three assays and include the diameter of the wells (6 mm).

**Tab. 3:** Susceptibility of *Lactobacillus* strains to antibiotics by disc diffusion test

	Inhibition Diameters (mm)^a^				

Strains	AMP_10_	ERY_15_	PEN_10_	CHL_30_	TET_30_
***Lb. plantarum 2S***	28 (S)	30 (S)	23 (S)	22 (S)	22 (S)
***Lb. plantarum 5S***	31 (S)	27 (S)	22 (S)	20 (I)	20 (I)
***Lb. plantarum 6S***	30 (S)	30 (S)	23 (S)	25 (S)	26 (S)
***Lb. plantarum 7S***	28 (S)	30 (S)	27 (S)	25 (S)	23 (S)
***Lb. plantarum 8S***	31 (S)	28 (S)	30 (S)	37 (S)	22 (S)
***Lb. plantarum 9S***	34 (S)	30 (S)	25 (S)	25 (S)	25 (S)
***Lb. plantarum 10S***	28 (S)	26 (S)	21 (S)	25 (S)	20 (I)
***Lb. plantarum 11S***	28 (S)	27 (S)	22 (S)	25 (S)	18 (I)
***Lb. plantarum 16S***	27 (S)	27 (S)	20 (I)	27 (S)	22 (S)
***Lb. rhamnosus 18S***	35 (S)	30 (S)	22 (S)	27 (S)	29 (S)
***Lb. rhamnosus 1K***	35 (S)	32 (S)	24 (S)	22 (S)	25 (S)
***Lb. fermentum 2K***	27 (S)	28 (S)	26 (S)	25 (S)	21 (S)

	**Inhibition Diameters (mm)**^a^				

	**GEN_30_**	**CIP_10_**	**AMO_10_**	**COT_25_**	**DOX_30_**

***Lb. plantarum 2S***	13 (R)	13 (R)	30 (S)	22 (S)	21 (S)

***Lb. plantarum 5S***	13 (R)	7 (R)	30 (S)	17 (I)	20 (I)

***Lb. plantarum 6S***	11 (R)	10 (R)	28 (S)	20 (I)	26 (S)

***Lb. plantarum 7S***	12 (R)	12 (R)	30 (S)	22 (S)	22 (S)

***Lb. plantarum 8S***	14 (R)	12 (R)	28 (S)	25 (S)	24 (S)

***Lb. plantarum 9S***	22 (S)	14 (R)	33 (S)	20 (I)	29 (S)

***Lb. plantarum 10S***	16 (I)	7 (R)	25 (S)	23 (S)	23 (S)

***Lb. plantarum 11S***	18 (I)	12 (R)	26 (S)	21 (S)	25 S)

***Lb. plantarum 16S***	14 (R)	11 (I)	28 (S)	20 (I)	25 (S)

***Lb. rhamnosus 18S***	21 (S)	14 (R)	32 (S)	18 (I)	28 (S)

***Lb. rhamnosus 1K***	20 (I)	15 (R)	29 (S)	16 (I)	26 (S)

***Lb. fermentum 2K***	16 (I)	11 (R)	25 (S)	24 (S)	26 (S)

AMO_10_: Amoxicillin 10μg; AMP_10_: Ampicillin 10μg; COT_25_: Co-trimoxazole (Trimethoprim/Sulfamethoxazole) 1.25+23.75= 25μg; CHL_30_: Chloramphenicol 30μg; CIP_10_: Ciprofloxacin 10 μg; DOX_30_: Doxycyclin 30μg; ERY_15_: Erythromycin 15μg; PEN_10_: Penicillin G 10μg; TET_30_: Tetracycline 30 μg; GEN_30_: Gentamicin 30μg (CLSI, 2011).

a …Inhibition Zone Diameters are means from triplicate determinations; Diameters of the discs are inclusive (6mm); S…Sensitive; I…Intermediate; R…Resistant.

**Tab. 4:** MIC values (in μg/ml) of antibiotics against *Lactobacillus* strains.

Strains	AMP		PEN		ERY		CHL		TET	

	MIC μg/ml	MIC BP*	MIC μg/ml	MIC BP*	MIC μg/ml	MIC BP*	MIC μg/ml	MIC BP*	MIC μg/ml	MIC BP*
*Lb. plantarum* 2S	0.5	2	2	ND	<0.25	1	2	8	8	32
*Lb. plantarum* 5S	0.5	2	2	ND	<0.25	1	2	8	8	32
*Lb. plantarum* 6S	0.5	2	1	ND	<0.25	1	2	8	8	32
*Lb. plantarum* 7S	0.5	2	1	ND	<0.25	1	2	8	16	32
*Lb. plantarum* 8S	1	2	2	ND	<0.25	1	2	8	8	32
*Lb. plantarum* 9S	1	2	1	ND	<0.25	1	4	8	16	32
*Lb. plantarum* 10S	0.5	2	1	ND	<0.25	1	2	8	8	32
*Lb. plantarum* 11S	0.5	2	1	ND	<0.25	1	4	8	8	32
*Lb. plantarum* 16S	0.5	2	1	ND	<0.25	1	2	8	16	32
*Lb. rhamnosus* 18S	0.5	4	1	ND	<0.25	1	2	4	8	8
*Lb. rhamnosus* 1K	0.5	4	2	ND	<0.25	1	2	4	16	8
*Lb. fermentum* 2K	0.5	1	1	ND	<0.25	1	2	4	8	8

	**GEN**		**AMO**		**CIP**		**COT**		**DOX**	
	
	MIC μg/ml	MIC BP*	MIC μg/ml	MIC BP*	MIC μg/ml	MIC BP*	MIC μg/ml	MIC BP*	MIC μg/ml	MIC BP*

*Lb. plantarum* 2S	64	16	1	ND	64	ND	32	ND	1	ND
*Lb. plantarum* 5S	64	16	0.5	ND	128	ND	32	ND	2	ND
*Lb. plantarum* 6S	64	16	1	ND	64	ND	32	ND	2	ND
*Lb. plantarum* 7S	64	16	0.5	ND	64	ND	64	ND	4	ND
*Lb. plantarum* 8S	64	16	0.5	ND	64	ND	64	ND	4	ND
*Lb. plantarum* 9S	64	16	1	ND	64	ND	64	ND	4	ND
*Lb. plantarum* 10S	32	16	0.5	ND	128	ND	64	ND	4	ND
*Lb. plantarum* 11S	64	16	0.5	ND	64	ND	32	ND	4	ND
*Lb. plantarum* 16S	64	16	2	ND	64	ND	32	ND	4	ND
*Lb. rhamnosus* 18S	64	16	0.5	ND	64	ND	32	ND	4	ND
*Lb. rhamnosus* 1K	64	16	0.5	ND	64	ND	64	ND	4	ND
*Lb. fermentum* 2K	64	16	0.5	ND	64	ND	64	ND	2	ND

* MIC BP = Minimal Inhibitory Concentration Breakpoints, according to European Food Safety Authorities [5]. ND…Not Defined.

**Tab. 5:** Antibacterial spectrum of activity of bacteriocins produced by selected strains.

			Bacteriocin Activity of Producer strains											
			
Indicator strains	Source	Growth conditions	2S	5S	6S	7S	8S	9S	10S	11S	16S	18S	1K	2K
**lactic acid bacteria**														

*Lb. plantarum 3S*	Our isolate	MRS^c^,30°C	++	++	+++	++	++	++	+	++	++	++	++	++
*Lb. plantarum 9S*	Our isolate	MRS,30°C	−	−	−	−	−	−	−	−	−	−	−	−
*Lb. plantarum 29V*	Our collection	MRS,30°C	−	−	−	−	−	−	−	−	−	−	−	−
*Lb. rhamnosus 18S*	Our isolate	MRS,30°C	−	−	−	−	−	−	−	−	−	−	−	−
*Lb. rhamnosus 1K*	Our isolate	MRS,30°C	−	−	−	−	−	−	−	−	−	−	−	−
*Lb. fermentum 2K*	Our isolate	MRS,30°C	−	−	−	−	−	−	−	−	−	−	−	−
*Enterococcus faecium*	DSM^a^ 13596	BHI^d^,37°C	−	−	−	−	−	−	−	−	−	−	−	−

**Gram-positive pathogenic bacteria**														

*Staphylococcus aureus*	ATCC 25923	BHI, 37°C	++	++	++	++	++	++	++	++	++	++	++	++
*Staphylococcus aureus (MDR)*	Clinical isolate^f^	BHI, 37°C	++	++	++	++	++	++	++	++	++	++	++	++
*Bacillus cereus*	ATCC 11778	BHI, 37°C	++	++	++	++	++	++	++	++	++	++	++	++
*Streptococcus mutans*	DSM 20523	BHI, 37°C	++	+++	+++	++	++	++	++	++	+++	++	+++	+++

**Gram-negative pathogenic bacteria**														

*Escherichia coli*	ATCC 11775	BHI, 37°C	++	++	++	++	++	+++	++	++	++	++	++	++
*Escherichia coli (MDR)*	Clinical isolate	BHI, 37°C	−	−	+	−	−	−	−	−	−	−	++	−
*Salmonella* Typhi	ATCC 6539	NBe, 37°C	+++	+++	+++	++	+++	+++	+++	+++	+++	+++	+++	+++
*Pseudomonas aeruginosa*	ATCC 9027	BHI, 37°C	+++	++	+++	+++	++	++	++	++	+++	++	+++	++
*Pseudomonas aeruginosa*	ATCC 27853	BHI, 37°C	++	++	++	++	++	++	++	++	++	++	++	++
*Klebsiella pneumoniae*	Clinical isolate	BHI, 37°C	++	++	+++	++	++	++	++	++	+++	++	++	++
*Shigella flexneri*	Clinical isolate	NB, 37°C	+	+	++	++	++	+++	++	++	+++	++	++	+++

Inhibition zone Diameter: +++ = >14mm; ++ = 11–14mm; + = 8–10mm; − = no inhibition. MDR: Multidrug-resistant;

a: DSM: Deutsche Sammlung von Mikroorganismen und Zellkulturen GmbH, Braunschweig, Germany;

b: ATCC: American Type Culture Collection, Manassas, VA, USA;

c: de Man, Rogosa and Sharpe;

d: Brain Heart Infusion;

e: Nutrient Broth;

f: Isolates from Centre Pasteur of Yaoundé, Cameroon.
